# Ocular findings in patients with spastic type cerebral palsy

**DOI:** 10.1186/s12886-016-0367-1

**Published:** 2016-11-08

**Authors:** Myung Jin Park, Yung Ju Yoo, Chin Youb Chung, Jeong-Min Hwang

**Affiliations:** 1 Department of Ophthalmology, Seoul National University College of Medicine, Seoul National University Bundang Hospital, 166, Gumiro, Bundang-gu, Seongnam, Gyeonggi-do 463-707 Korea; 2 Department of Orthopaedic surgery, Seoul National University College of Medicine, Seoul National University Bundang Hospital, 166, Gumiro, Bundang-gu, Seongnam, Gyeonggi-do 463-707 Korea

**Keywords:** Spastic type, Cerebral palsy, Ocular findings, Strabismus

## Abstract

**Background:**

Refractive errors, strabismus, nystagmus, amblyopia, and cortical visual impairment are observed in 50 to 90 % of patients with cerebral palsy. Ocular abnormalities are known to differ according to cerebral palsy type, and spastic type has been reported to be more likely to be associated with ocular defects than the athetoid and ataxic types.

**Methods:**

A retrospective review of medical records was performed on 105 consecutive children with spastic type of cerebral palsy who underwent ophthalmologic examination between July 2003 and March 2006. The complete ophthalmological examination included measurement of visual acuity, ocular motility, stereoacuity, binocular vision, cycloplegic refraction along with the evaluation of the anterior segment and the posterior segment.

**Results:**

The most common ocular abnormality was strabismus (70.5 %) followed by refractive errors (53.3 %). Exodeviation was more commonly found than esodeviation (46 vs 27 patients), and hyperopia was much more prevalent than myopia. A considerable number of patients with strabismus had abnormal ocular motility wherein 16 patients showed inferior oblique overaction and ten superior oblique overaction. Whereas inferior oblique overaction was accompanied similarly in exotropia and esotropia, superior oblique overaction was accompanied more by exotropia.

**Conclusions:**

Children with spastic type cerebral palsy have a high prevalence of strabismus and refractive errors. Exotropia and hyperopia are the most common ocular abnormalities. All children with spastic type of cerebral palsy may require a detailed ophthalmologic evaluation.

## Background

Cerebral palsy is a disorder of movement and posture due to a defect or lesion in the immature brain [1, 2]. Cerebral palsy can be classified as spastic (four subtypes), ataxic, athetoid, or atonic type according to motor abnormalities [1, 2]. Ophthalmological problems including significant refractive errors, strabismus, nystagmus, and amblyopia as well as cortical visual impairment are observed in 50–90 % of the patients with cerebral palsy [3–17]. Ocular abnormalities are known to differ according to cerebral palsy type [3, 4]. Among the four types, spastic type is more commonly associated with ocular abnormalities than athetoid or ataxic types [5–11]. For this reason, it may be important to determine detailed ocular findings in spastic type of cerebral palsy. However, there are limited data on studies examining ocular abnormalities in the spastic type of cerebral palsy [12]. Therefore, we investigated ocular findings, such as refractive errors, strabismus, and dysfunction of oblique muscles associated with strabismus in spastic type of cerebral palsy.

## Methods

Approval to conduct this study was obtained from the Institutional Review Board. We undertook a retrospective analysis of 216 patients with cerebral palsy who had visited the Department of Ophthalmology at Seoul National University Bundang Hospital from July 2003 to March 2006, and underwent a full ophthalmologic examination including evaluation of the visual acuity, ocular motility, stereoacuity and binocular vision, cycloplegic refraction with cyclopentolate hydrochloride (1 %), slit lamp examination, and fundus examination by one author (J-MH).

Fifty-five patients who underwent previous strabismus surgery and patients that did not undergo a full ophthalmologic examination including a detailed motility examination were excluded. Fifty-six patients with types of cerebral palsy other than spastic type were also excluded. One author (CYC) made the diagnosis of cerebral palsy and classified the sub-type of cerebral palsy.

One hundred and five patients were enrolled in this study. Sixty-five were male and 40 were female. Age at first ophthalmologic examination ranged from 2 to 22 years (mean 6.6 years), and mean follow up duration was 8.7 months (range, 1–34 months).

Visual acuities were evaluated as follows: patients were seated at 6 m from the chart, which was positioned at eye level. Each eye was occluded in turn and visual acuity was measured monocularly using the Snellen visual chart. In patients who were unable to identify letters verbally in spite of the repeated instruction and encouragement, visual fixation was assessed with approximately 5-in. interesting object at near as of quality and accuracy (good, fair, poor), location (central vs eccentric), and duration (maintained vs sporadic). Then, fixation target was slowly moved though the visual field to assess the quality of fixation on following.

Refractive errors of spherical equivalent of +1.50 diopters or more, or −1.50 diopters or less, or astigmatism of 1.50 diopters or more were defined to be clinically significant hyperopia, myopia and astigmatism, respectively. Anisometropia was defined as ≥1.0 diopters spherical equivalent difference between eyes. Prism and alternate cover test with accommodative targets for fixation at 1/3 and 6 m were conducted ​in most of the patients and a modified Krimsky test at 1/3 m was also performed in a few uncooperative patients. In patients who showed distance-near disparity in the measurement of exodeviation, an additional near measurement was obtained after 1 h of monocular occlusion of the deviating eye, and another postocclusion near measurement was obtained with an additional +3.00 diopters sphere over each eye prior to allowing the patient to regain binocular fusion. Sensory status was evaluated using the Titmus stereotest and the Worth 4-dot test at distance and near in cooperative patients. Ductions and versions including associated abnormalities of oblique muscles were carefully evaluated. The prevalence of oblique muscle dysfunction in exotropia and esotropia were compared using the Chi-square test. *P* values of < 0.05 were considered significant, and all analyses were performed using SPSS for Windows Version 11.0.0 (SPSS Inc., Chicago, IL). Patients with a difference of two lines or more of best corrected visual acuity in each eye without any other diseases such as retinal or optic nerve disease, glaucoma, cataract, nystagmus, media opacities, and ocular trauma were considered to have amblyopia.

## Results

Among the subtypes of spastic type of cerebral palsy, diplegia was most common followed by hemiplegia and tetraplegia (Table 1). Sixteen (69.6 %) out of 23 patients who underwent brain imaging had periventricular leukomalacia (Table 1). The associated perinatal defects occurring in this series were prematurity in 51, seizures in four, hypoxia in two, and hydrocephalus in two patients (Table 1).

**Table 1:** Clinical characteristics of patients with spastic type of cerebral palsy

	No of patients	Percent (%)
Incidence of the subtypes of spastic type of cerebral palsy		
Diplegia	47	44.8
Hemiplegia	27	25.7
Tetraplegia	17	16.2
Monoplegia	1	1.0
Unclassified	13	12.3
Total	105	100
Associated brain abnormalities		
Periventricular leukomalacia	16	69.6
Brain atrophy	2	8.4
Brainstem hemiatrophy	1	4.4
Frontal lobe calcification	1	4.4
Diffuse atrophy of cerebellum	1	4.4
Corpus callosum agenesis	1	4.4
Normal finding	1	4.4
Total	23	100
Associated perinatal defects		
Prematurity	51	81.0
Seizure	4	6.4
Hypoxia	2	3.2
Hydrocephalus	2	3.2
Premature, hypoxia	2	3.2
Hypoxia, brain hemorrhage	1	1.5
Premature, seizure	1	1.5

Best corrected visual acuity was variable ranging from no light perception to 20/20. Best corrected visual acuity corresponded to the mild vision loss < 0.8 and ≥ 0.3 in 33 eyes in the right and in 32 eyes in the left (Table 2). In addition, four patients showed no fix and follow in either eye. Poor fix and follow was observed in five right eyes and four left eyes, and good fix and follow, in 41 right eyes and 42 left eyes. The best corrected visual acuity of the better eye was mildly impaired of < 0.8 and ≥ 0.3 in 29 patients, moderately impaired of < 0.3 and ≥ 0.125 in five patients, severely impaired of < 0.125 and ≥ 0.05 in five patients. Strabismus was found in 74 patients (70.5 %). Among the 74 patients with strabismus, 46 patients had an exodeviation, 27 esodeviation and 1 dissociated vertical deviation (Table 3). Of the 46 patients with exodeviation, four had exophoria, 20 intermittent exotropia, while the remainder had constant exotropia. The amount of exodeviation was ≤ 10 prism diopters in five patients, 11–20 prism diopters in 19 patients, 21–30 prism diopters in 13 patients, 31–40 prism diopters in six patients and over 40 prism diopters in three patients. Only one of the 27 patients with esodeviation had accommodative esotropia; the remainder had non-accommodative esotropia. The amount of esodeviation was ≤ 10 prism diopters in two patients, 11–20 prism diopters in six patients, 21–30 prism diopters in five patients, 31–40 prism diopters in eight patients and over 40 prism diopters in six patients. In patients with significant strabismus which was not corrected with glasses as well as occlusion for amblyopia, strabismus surgery was recommended and was performed in some patients.

**Table 2:** Best corrected visual acuity in patients with spastic type of cerebral palsy

Visual acuity	Right eye	Left eye
Normal vision ≥ 0.8	7	5
Mild vision loss < 0.8 and ≥ 0.3	33	32
Moderate vision loss < 0.3 and ≥ 0.125	11	12
Severe vision loss < 0.125 and ≥ 0.05	4	5
Profound vision loss < 0.05 and ≥ 0.02	0	0
Near-total vision loss < 0.02 and ≥ NLP	0	0
Total vision loss (NLP)	0	1

**Table 3:** Ocular alignment in patients with spastic type of cerebral palsy

	No of patients	Percent (%)
Exotropia	33	31.4
Esotropia	25	23.9
Exotropia + DVD	5	4.8
Exophoria	4	3.8
Exotropia + hypertropia	3	2.7
Esotropia + DVD	2	1.9
DVD	1	1.0
Exotropia + hypertropia + DVD	1	1.0
No strabismus	31	29.5
Total	105	100

*DVD* Dissociated vertical deviation

Significant refractive errors were found in 56 patients (53.3 %); 19 hyperopia ≥ +1.50 diopters (mean ± standard deviation (SD), +2.86 ± 1.30 diopters), seven myopia ≥ −1.50 diopters (mean ± SD, −3.78 ± 2.24 diopters), 21 hyperopic astigmatism ≥ +1.50 diopters (mean ± SD, +2.46 ± 1.90 diopters) and nine myopic astigmatism ≥ −1.50 diopters (mean ± SD, −1.68 ± 0.91 diopters). Anisometropia ≥ 1.00 diopter was found in 24 patients (mean ± SD, 2.36 ± 2.49 diopters). Anisometropia ≥ 2.00 diopters was found in seven patients (mean ± SD, 4.64 ± 3.68 diopters). In patients with significant refractive errors, glasses were prescribed. Prescription of glasses depends on the level of visual acuities in each eye, the degree of anisometropia and astigmatism, the state of strabismus as well as patients age. The criteria for the prescription of glasses weremore than +3.00 D, myopia and astigmatism more than −1.50 D In patients with esotropia, exceptively, any hyperopia ≥ +1.00 D was fully corrected, as was those with exotropia, any myopia ≤ −1.00 D was fully corrected. Both uncorrected visual acuity and best corrected visual acuity were measured in 18 patients. Visual acuity was significantly improved in both eyes (*P* = 0.003 and 0.001 respectively for right and left eye, paired T-test, Table 4).

**Table 4:** Comparison of uncorrected visual acuity and best-corrected visual acuity in patients with spastic cerebral palsy

	UCVA	BCVA	*P* value*
Visual acuity, Right eye	0.33 ± 0.22	0.55 ± 0.23	0.003
Visual acuity, Left eye	0.26 ± 0.18	0.46 ± 0.25	0.001

* *P* value by paired T-test

Data are mean ± standard deviation

*BCVA* Best corrected visual acuity, *UCVA* Uncorrected visual acuity

Sixteen patients showed inferior oblique overaction, two inferior oblique underaction, ten superior oblique overaction, and three superior oblique underaction (Table 5). The incidence of inferior oblique muscle overaction was similar both in patients with exotropia and esotropia (*P* = 0.203), and that of the superior oblique overaction was found more in patients with exotropia than in esotropia, but without significance (*P* = 0.18) (Table 5). In addition, mild abduction limitation was found in patient with esotropia, and another patient with esotropia was found to have upgaze limitation.

**Table 5:** Abnormalities of oblique muscles according to types of strabismusin patients with spastic type of cerebral palsy

	IOOA	IOUA	SOOA	SOUA
Exotropia	7	2	8	2
Esotropia	8	0	2	1
DVD	1	0	0	0

*IOOA* inferior oblique overaction, *IOUA* inferior oblique underaction, *SOOA* superior oblique overaction, *SOUA* superior oblique underaction, *DVD* dissociated vertical deviation

Of 25 patients who could perform Titmus stereotest, nine patients showed stereoacuity of 3000 s of arc, five patients 400 s of arc, two patients 200 s of arc, seven patients 100 s of arc, one patient 80 s of arc, and one patient 60 s of arc. Logistic regression analyses showed no correlation between age and stereoacuity (OR = 0.988, *P* = 0.852).

Of 55 patients who could perform Worth four dot test, 14 patients showed the fusional response both at distance and near and 20 patients showed suppression both at distance and near. Rest of the patients showed various responses (Table 6).

**Table 6:** Responses of Worth 4 dot test

	Fusion at near	Suppression at near	Diplopia at near
Fusion at distance	14	4	7
Suppression at distance	4	20	0
Diplopia at distance	0	5	1

Slit lamp examination showed corneal erosions in six patients, papillary hypertrophy in four patients, mild lens opacity in one patient and no abnormal findings in the rest of the patients. The causes of corneal erosions included epiblepharon in the lower eyelid in three patients, dry eye and allergic conjunctive in one patient, incomplete lid closure in one patient, and indefinite in one remaining patient. One patient underwent a repair of epiblepharon. Fundus examination revealed optic disc pallor in nine patients, tigroid fundus in two patients, tilted disc in one patient, asymmetric disc cupping in one patient and no abnormal findings in the rest of the patients.

There was no differences in age, best corrected visual acuity, and spherical equivalent among subtypes of spastic cerebral palsy (diplegia, hemiplegia, or tetraplegia) (Table 7). In addition, incidence of horizontal diplopia type, presence of vertical diplopia, and presence of dissociated vertical deviation was similar regardless of subtypes of spastic palsy (Table 7).

**Table 7:** Clinical characteristic according to subtype of spastic type of cerebral palsy

	Diplegia (*N* = 47)	Hemiplegia (*N* = 27)	Tetraplegia (*N* = 17)	*P* value
Age (years)	6.9 ± 3.8 (2,19)	6.9 ± 4.7 (2,22)	6.8 ± 4.1 (2,17)	0.997*
F/U duration (month)	8.9 ± 8.8 (1,31)	8.9 ± 8.6 (1,32)	8.4 ± 9.6 (1,34)	0.975*
Visual acuity (LogMAR) OD	0.42 ± 0.31 (0,1.00)	0.42 ± 0.25 (0.05,0.82)	0.42 ± 0.24 (0,0.70)	0.978*
Visual acuity (LogMAR) OS	0.48 ± 0.31 (0.05,1.30)	0.44 ± 0.25 (0.10,0.82)	0.45 ± 0.26 (0,0.70)	0.890*
Spherical equivalent, OD	0.20 ± 3.34 (−16.0,6.0)	0.07 ± 2.39 (−6.75,4.25)	−0.87 ± 3.52 (−9.00,3.75)	0.472*
Spherical equivalent, OS	0.52 ± 2.42 (−6.25,5.50)	0.51 ± 2.80 (−6.00,9.00)	0.10 ± 2.87 (−5.75,5.00)	0.842*
Presence of prematurity (n,%)	31 (64.6 %)	10 (37.0 %)	8 (44.4 %)	0.053†
Presence of seizure (n,%)	3 (6.3 %)	1 (3.7 %)	1 (5.6 %)	0.311†
Presence of PVL (n,%)	10 (76.9 %)	3 (42.9 %)	1 (60.0 %)	
Horizontal strabismus				
Orthotropia (n,%)	12 (25.5 %)	7 (25.9 %)	5 (27.8 %)	0.983†
Exotropia (n,%)	22 (46.8 %)	12 (44.4 %)	7 (38.9 %)	0.848†
Esotropia (n,%)	12 (25.5 %)	7 (25.9 %)	5 (27.8 %)	0.983†
Exophoria (n,%)	1 (2.1 %)	1 (3.7 %)	1 (5.6 %)	0.775†
Vertical diplopia (n,%)	1 (2.1 %)	3 (11.1 %)	1 (5.2 %)	0.114†
Presence of DVD (n,%)	5 (10.6 %)	2 (8.0 %)	1 (11.1 %)	0.924†

*OD* Right eye, *OS* Left eye, *PVL* periventricular leukomalacia

* *P* value by ANOVA

† *P* value by Chai square test

## Discussion

Previous studies about cerebral palsy have reported variable incidence of ocular abnormalities including significant refractive errors, strabismus, nystagmus, amblyopia, cortical visual impairment and so on [3–16]. Among them, significant refractive errors and strabismus were most commonly reported [3–16].

Our study revealed that 56 % of the patients with spastic type of cerebral palsy had significant refractive errors and 71 % strabismus. There was another study to evaluate the ocular findings confined to children with spastic type of cerebral palsy. Ozturk et al. [12] reported ocular abnormalities of significant refractive errors ≥ ±1.00 diopter (70.1 %), strabismus (55.2 %), abnormal optic disc (39.2 %), nystagmus (18.6 %), and anisometropia >1.00 diopter (14.4 %), which are comparable to our study. Two different findings from the previous reports include, firstly, exodeviation was more commonly found than esodeviation. Secondly, superior oblique overaction is frequently accompanied by exotropia, whereas inferior oblique overaction is accompanied similarly by exotropia and esotropia, which has not been described.

Disease pathology in spastic type of cerebral palsy is more extensive and diffuse than athetoid and ataxic types, and is associated with periventricular hemorrhage, subcortical hemorrhage, and cortical atrophy [11]. The optic radiation axons pass through the periventricular space, thus visual function also could be affected with periventricular leukomalacia in cerebral palsy [17]. For this reason, it is important to determine detailed ocular findings in spastic type of cerebral palsy. As far as we are aware, the present study includes the largest number of spastic type of cerebral palsy patients.

Regarding refractive errors in cerebral palsy, 40 to 50 % of cerebral palsy patients have been reported to have refractive errors [13, 14]. Fantl and Perlstein [13] noted that cerebral palsy patients are at greater risk of hyperopia than normal subjects. Moreover, as patient age increases, the degree of hyperopia tended to remain constant in contrast to the progression of myopia. Hyperopia is known to be related to athetoid type rather than spastic type, and to be more related to cerebral palsy caused by hypoxia rather than prematurity [13]. In contrast, myopia predominates in the spastic group. Lo Cascio [9] noted that spastic diplegia in particular carried a high risk of refractive errors and athetoid cerebral palsy the least risk. In this study, which included 105 spastic type patients, hyperopia was 2.5 fold more prevalent than myopia. This is all the more surprising when one considers the prevalence of myopia compared with that of hyperopia in Asians.

Whereas Caucasians without cerebral palsy have an 8–10:1 ratio of esotropia to exotropia, Caucasian cerebral palsy patients have a ratio of 1.9:1 or 2–3:1 [6, 8, 15]. In Korea, it has been reported that exotropia occurs 5.8 times more than esotropia in the normal population [18]. In the present study, exotropia was also found to be more common than esotropia, but at a much lower frequency than in normal subjects, i.e., exotropia was found to occur 1.5 times more than esotropia.

Another issue that deserves attention is that superior oblique overaction was frequently accompanied by exotropia, whereas inferior oblique overaction was accompanied similarly by exotropia and esotropia. Moreover, no report has described the dysfunction of oblique muscles in cerebral palsy, and thus this result stands alone. However, these findings on oblique overaction were not statistically significant.

Even though the present study was conducted on a larger number of spastic type of cerebral palsy patients than prior studies, and presents a detailed description of strabismus and oblique muscle dysfunction, it is limited in that it included patients with spastic type of cerebral palsy who visited a referral hospital and the results are only in regards to an Asian population referred for ophthalmologic care.

Regarding the impact of visual impairment associated with cerebral palsy to the quality of life, Boyaci et al. [19] found no significant correlations between the quality of life and fundus pathology or strabismus. However, they did not assessed the visual acuity. In contrast, Tessier et al. [20] reported that visual impairment could be a strong negative impact on psychosocial quality of life in children with cerebral palsy. Mitry et al. [21] also showed a strong association of perceptual visual dysfunction with total as well as emotional/social quality of life scores. Problems with visual attention/recognition/navigation were associated with lower total as well as emotional/social quality of life scores, and those with visual search, with emotional/social quality of life scores. We did not evaluate the questionnaires of the quality of life.

There is a limitation in our study. Our hospital is the tertiary one, and the patients visited our clinic with the recognition of the ophthalmologic problems or were recommended to visit our clinic by the orthopedic surgeons who fully recognize the necessity of the ophthalmologic examination. Therefore, our study may overestimate the prevalence of ophthalmologic problems in the general population of cerebral palsy.

## Conclusion

In conclusion, the prevalence of refractive errors and strabismus was considerably higher in patients with spastic type of cerebral palsy than in general population. Exotropia and hyperopia are the most common type of strabismus and refractive errors, respectively. There was no differences in clinical and ocular characteristics among subtypes of spastic cerebral palsy. Our results indicate that all children with spastic type of cerebral palsy may require a detailed ophthalmologic evaluation.
